# Folate Deficiency Alters microRNA Expression and Transcriptomic Networks in a Human Trophoblast Model

**DOI:** 10.3390/nu18172785

**Published:** 2026-08-26

**Authors:** Bernadette C. Baker, Georgia Fakonti, Abigail R. Byford, Fiona L. Mackie, Samantha C. Lean, Ainslie Garrod, Lucy Poffley, Leo A. H. Zeef, Susan L. Greenwood, Alexander E. P. Heazell, Rebecca L. Jones, Karen Forbes

**Affiliations:** 1 Maternal and Fetal Health Research Centre, Division of Developmental Biology and Medicine, School of Medical Sciences, Faculty of Biology, Medicine and Health, University of Manchester, Manchester M13 9WL, UK; fiona.mackie1@nhs.net (F.L.M.); s.sansby1228@gmail.com (S.C.L.); ainslie.garrod@manchester.ac.uk (A.G.); lucy.poffley@doctors.net.uk (L.P.); susan.l.greenwood@manchester.ac.uk (S.L.G.); alexander.heazell@manchester.ac.uk (A.E.P.H.); rebeccaheazellnutrition@gmail.com (R.L.J.); 2 St Mary’s Hospital, Manchester University NHS Foundation Trust, Manchester Academic Health Science Centre, Manchester M13 9PL, UK; 3 Discovery and Translational Science Department, Leeds Institute of Cardiovascular and Metabolic Medicine, University of Leeds, Leeds LS2 9JT, UK; umgf@leeds.ac.uk (G.F.); abigail.byford@manchester.ac.uk (A.R.B.); 4 Department of Obstetrics and Gynaecology, The Royal Wolverhampton NHS Trust, New Cross Hospital, Wolverhampton WV10 0QP, UK; 5 Bioinformatics Core Facility, University of Manchester, Manchester M13 9PT, UK; leo.zeef@manchester.ac.uk

**Keywords:** nutrition, folate, placenta, microRNA, functional enrichment analysis

## Abstract

**Background:** Low maternal folate status is associated with placental dysfunction and adverse pregnancy outcomes; however, the mechanisms linking reduced folate availability to altered placental function remain incompletely understood. We investigated whether folate deficiency directly alters trophoblast function and microRNA (miRNA) expression, and whether folate-responsive miRNAs mediate these functional changes. **Methods and Results:** Human placental villous explants, BeWo choriocarcinoma cells, and primary human cytotrophoblasts were cultured under physiological or folate-deficient conditions to assess the direct impact of reduced folate availability. Although intracellular folate depletion was achieved in all models, only primary cytotrophoblasts reproduced functional changes consistent with those observed in placentas from folate-deficient pregnancies, exhibiting increased apoptosis and reduced system A amino acid transport. Of sixteen miRNAs previously associated with low maternal folate status, miR-30e-3p and miR-34b-5p were significantly reduced in trophoblast following folate depletion. Targeted inhibition of either miRNA did not alter apoptosis or system A activity. Pathway analysis of differentially expressed genes following miRNA inhibition identified processes related to cytoskeletal organisation, cell adhesion, PI3K/AKT and MAPK signalling. **Conclusions:** Folate deficiency directly impairs trophoblast survival, amino acid transport, and miRNA expression in primary trophoblasts. Our findings demonstrate that only a subset of folate-associated placental miRNAs respond directly to folate depletion and that inhibition of individual folate-responsive miRNAs is insufficient to reproduce the trophoblast phenotype. These results indicate that trophoblast adaptation to reduced folate availability is likely mediated through coordinated nutrient-sensitive regulatory networks rather than individual miRNAs acting in isolation.

## 1. Introduction

Folate (vitamin B9) is a critical micronutrient required for nucleotide biosynthesis and one-carbon metabolism and is therefore particularly important during periods of rapid cell growth and differentiation, including pregnancy [1]. As folate cannot be synthesised endogenously, maternal folate status depends on dietary intake and supplementation [2,3,4]. Low folate status during pregnancy is associated with adverse birth outcomes, most notably neural tube defects (NTDs) and impaired fetal growth [3,5,6,7,8]. Although periconceptional supplementation reduces NTD risk and improves placental and fetal growth [9,10,11,12] folate deficiency remains common globally, highlighting the need to better understand its biological consequences [13,14,15].

The placenta plays a central role in mediating the effects of maternal folate status on fetal development. Folate transfer across the maternal–fetal interface is facilitated by specific placental folate transporters [16], and the placenta undergoes rapid growth and functional adaptation throughout gestation, rendering it particularly sensitive to nutritional perturbation [17,18,19,20]. Both human and animal studies demonstrate that folate deficiency is associated with placental dysfunction, including altered trophoblast cell turnover, endocrine function and amino acid transport [21,22,23]. In vitro studies using trophoblast-derived cell lines and primary trophoblasts further support a direct effect of folate availability on placental function and development [23,24,25,26,27].

The molecular mechanisms linking folate deficiency to placental dysfunction remain incompletely understood. MicroRNAs (miRNAs) regulate trophoblast proliferation, survival, differentiation and nutrient transport [28,29,30,31], and are dysregulated in several pregnancy complications associated with impaired fetal growth [32,33]. We have previously reported altered placental miRNA expression in association with low maternal folate status [21], and similar findings are reported in animal models [34]. However, these observational studies are largely associative, and it remains unclear whether altered miRNA expression is a direct consequence of folate deficiency and whether such changes contribute functionally to placental dysfunction.

In the present study, we developed ex vivo and in vitro human trophoblast models to examine the effects of folate deficiency on placental function and miRNA expression independent of maternal or fetal confounders. We first determined which experimental model most closely reproduced functional features of placental folate deficiency observed in vivo. We then examined whether miRNAs previously associated with low maternal folate status are directly responsive to reduced folate availability and whether these folate-responsive miRNAs contribute to trophoblast dysfunction using targeted miRNA inhibition.

## 2. Experimental Section

### 2.1. Human Placental Collection

Term placentas (37–42 weeks gestation) were obtained from uncomplicated singleton pregnancies following elective Caesarean section, with written, informed consent and ethical approval (North West Regional Ethics Committee; 08/H1010/55+5). Exclusion criteria included maternal BMI < 18.5 or >30 kg/m^2^; maternal age > 40 years; infant birthweight with individualised birth weight centile (IBC) < 10 or >90 (GROW software, Perinatal Institute, Birmingham, UK, Version 6.4); and maternal metabolic disease, infection, fetal growth restriction, high folate supplementation (5 mg/day) or congenital anomalies. All placentas were collected and sampled within 30 min of delivery following best practice guidelines [35,36].

### 2.2. Cell and Tissue Culture

#### 2.2.1. BeWo Choriocarcinoma Cells

BeWo choriocarcinoma cells (ATCC CCL-98) were cultured in folate-free RPMI-1640 media (FF-RPMI-1640; Gibco, Paisley, UK) containing 100 IU/mL penicillin, 100 µg/mL streptomycin sulphate, and 2 mM L-glutamine supplemented with 10% heat-inactivated Fetal Bovine Serum (FBS; Biosera, Rotherham, UK), resulting in final folate concentrations of 1 nM (deficient) or supplemented with folic acid (FA) to 25 nM (physiological). Cells were seeded at 50,000 cells/35 mm dish (Nunc, Fisher Scientific, Loughborough, UK) for system A activity or at 100,000 cells/well in 6-well plates for other analyses and cultured for 4–12 days depending on the endpoint.

#### 2.2.2. Human Placental Villous Explant Culture

Explant media was prepared using FF-RPMI1640 (Gibco, Paisley, UK) supplemented with 10% dialysed heat-inactivated FBS, penicillin (100 IU/mL), streptomycin (100 μg/mL), insulin (1 μg/mL), hydrocortisone (0.1 μg/mL), retinol acetate (0.1 μg/mL), calcium carbonate (1 mM), L-alanine (0.28 mM), L-cysteine (0.6 mM), and ascorbic acid (0.28 mM), adapted from Simán et al. [37]. Media were prepared at 0 nM (deficient) or 25 nM FA (physiological) and adjusted to pH 7.45. Full thickness villous biopsies (1 cm^3^) were collected using systematic uniform random sampling from 4 locations on the placenta [36]. Three explants (~5 mg each) were cultured in netwells (Corning, Fisher Scientific, Loughborough, UK) in folate-defined RPMI-1640 at 37 °C in a humidified chamber in a 5% CO_2_/95% air gas mixture. Conditioned media was collected daily for analysis and stored at −20 °C.

#### 2.2.3. Primary Human Cytotrophoblast Cells

Cytotrophoblasts were isolated by trypsin digestion and Percoll gradient separation as described previously [38]. Cells were plated in the same folate-defined media as BeWo cells at 1 nM or 25 nM FA into either 35 mm dishes at 2–3 × 10^6^ cells/dish for functional and molecular studies or 12-well plates (Corning, Appleton Woods, Birmingham, UK) containing sterile 16 mm diameter glass coverslips at a density of 1.5 × 10^6^ cells/well for immunohistochemistry and incubated in a humidified chamber at 37 °C in a 5% CO_2_/95% air–gas mixture. At 18 h after plating, cells were washed and fresh media added. Conditioned media were collected daily and stored at −20 °C. The folate-deplete medium (0–1 nM) was used as an in vitro model of folate deficiency to investigate the direct effects of reduced folate availability on placental function. The physiological concentration (25 nM) was selected to approximate serum folate concentrations in women with adequate folate status for prevention of NTDs as defined by the World Health Organisation (WHO) [39,40].

### 2.3. Intracellular Folate Measurement

Intracellular folate was extracted from explant tissue and cells using 0.5% ascorbic acid solution and quantified by microbiological assay (*Lactobacillus rhamnosus*). Briefly, tissue and cell samples were homogenised on ice using an Ultra-Turrax homogeniser (20,000 rpm, 30 s) and centrifuged at 10,000× *g* for 5 min at 4 °C. Folate concentrations were measured using a commercially available microbiological assay (ID-Vit Folic Acid; Immunodiagnostik AG, Bensheim, Germany). Sample turbidity was measured after 48 h at 37 °C at 630 nm on a Versamax plate reader (Molecular Devices, San Jose, CA, USA). Folate concentration (nmol/L) was corrected for explant wet weight (mg), cell number (BeWo cells) or total cell protein determined by the Bradford assay (trophoblast).

### 2.4. Analyses of Proliferation and Apoptosis

BeWo cell number (viable and non-viable) was determined after 4 days in culture using Trypan blue exclusion assay [41]. Proliferation and apoptosis in placental explants were assessed on formalin-fixed paraffin embedded (FFPE) sections (5 µm) by Ki67 as a marker of cells in cycle, and M30 immunohistochemistry, respectively, using specific primary antibodies (Ki76: 0.16 µg/mL, Dako; M30: 1 µg/mL, Roche Diagnostics, Burgess Hill, UK), or mouse IgG followed by goat anti-mouse biotinylated secondary (3.85 µg/mL, Dako, Agilent Technologies, Stockport, UK) as previously described [21]. In placental explants, the total number of cells was established by counting haematoxylin-positive nuclei and levels of proliferation and apoptosis were expressed as a percentage of total cells. Methanol-fixed primary trophoblasts were not assessed for proliferation because these cells undergo terminal differentiation in culture and were therefore analysed for apoptosis only, using M30 primary antibody (0.33 µg/mL, Roche Diagnostics, West Sussex, UK) and goat anti-mouse secondary antibody (2.57 µg/mL, Dako, Agilent Technologies, Stockport, UK). M30 staining was quantified using Histoquest Tissue Analysis software version 4.0 (TissueGnostics GmBH, Vienna, Austria) to determine the M30-positive cytoplasmic area relative to total (haematoxylin-positive) nuclear area.

### 2.5. Placental Endocrine Function

Levels of human chorionic gonadotrophin (hCG) in conditioned media from placental explant cultures were measured by ELISA (EIA-1469, DRG Diagnostics, Immunodiagnostics Systems Limited, Boldon Colliery, UK) following the manufacturer’s instructions. All data were normalised to explant weight. Intra-assay variation was 2.5–8.2% and inter-assay variation 10.6%. The more sensitive β-HCG ELISA (EIA-1911, DRG Diagnostics, Immunodiagnostics Systems Limited, Boldon Colliery, UK) was utilised for conditioned BeWo and trophoblast cell media and corrected for cell protein content using a Bio-Rad protein assay (Bio-Rad Laboratories, Hertfordshire, UK) as previously described [42]. Intra-assay variation was 0.9–8.5% and inter-assay variation 9.7%.

### 2.6. System A Transporter Activity

System A amino acid uptake was measured as the Na^+^-dependent uptake of ^14^C-methylaminoisobutyric acid (^14^C-meAIB) (Perkin Elmer, Buckinghamshire, UK), a non-metabolisable substrate of system A, normalised to cell protein content (measured by the Bio-Rad protein assay) and expressed as pmol ^14^C-meAIB uptake/mg protein as previously described [42]. System A activity was measured following 12 days of culture for BeWo cells, 4 days of culture for villous explants and 114 h of culture for primary isolated trophoblasts. Sodium-independent uptake and total cellular protein content were unchanged across conditions. 

### 2.7. RNA Isolation and miRNA Expression Analysis

Additionally, 10 ng of total RNA extracted using the mirVana miRNA isolation kit (Ambion, Life Technologies, Paisley, UK) was converted to cDNA using the miRCURY LNA Universal RT kit (Qiagen Ltd., Manchester, UK) following manufacturer’s protocol. miRNA expression was quantified by qPCR using individual miRCURY LNA assays (Supplementary Table S1; Qiagen Ltd., Manchester, UK) normalised to U6 snRNA, using a Stratagene MX3005P thermal cycler. Levels of miRNAs in folate-deficient conditions were expressed relative to physiological folate controls using the 2^(−ΔΔCt)^ method.

### 2.8. miRNA Inhibition Studies

Primary trophoblasts were transfected 18 h after plating, with mirVana inhibitors (40 nM; Thermo Fisher Scientific, Loughborough, UK) targeting hsa-miR-30e-3p or miR-34b-5p, or a non-targeting control (NTC), using Dharmafect 2 (Dharmacon, Horizon Discovery, Cambridge, UK) as previously described [43]. Functional and molecular endpoints were assessed 48 h post-transfection.

### 2.9. Transcriptomic and Pathway Analysis

RNA integrity assessment and gene-expression profiling were performed using Affymetrix Human Genome U133 Plus 2.0 arrays (Fisher Scientific, Loughborough, UK) by the Genomic Technologies Core Facility, University of Manchester. Differentially expressed genes (DEGs) were identified using PUMA software version 3.4.0 [44], with a probability of positive log ratio (PPLR) score < 0.1 or >0.9 and fold change < −1.5 or >1.5. Probe annotations were obtained using the Bioconductor package hgu133plus2.db (version 3.13.0) in R (version 4.3.1), and Affymetrix probe-set identifiers were mapped to corresponding gene annotations using AnnotationDbi (version 1.64.1). Probe sets lacking an annotated gene symbol were excluded. Where both _at and _s_at probe sets mapped to the same gene, the _at probe set was retained because of its greater target specificity. Where multiple _at probe sets mapped to the same gene, the mean log2 fold change was calculated and converted to fold change. Probe sets mapping to multiple genes and those with the Affymetrix _x_at designation, indicating potential cross-hybridisation, were excluded from downstream STRING, TargetScan and functional enrichment analyses. Gene Set Enrichment Analysis (GSEA) was performed separately using the complete transcriptomic dataset, including both differentially and non-differentially expressed genes. For GSEA, probes lacking gene annotations, probes mapping to multiple genes and _x_at probes were excluded. Where multiple probes mapped to the same gene, the probe with the strongest evidence for differential expression (PPLR furthest from 0.5) was retained. Genes were ranked by log2 fold change and analysed using fgsea (version 1.28.0) [45] against the human MSigDB Hallmark gene-set collection obtained using msigdbr (version 25.1.0) [46]. A false discovery rate (FDR)-adjusted *p* < 0.05 was considered statistically significant. Functional enrichment analysis of DEGs was performed using g:Profiler (version e114_eg62_p19_27110d83) [47]. Protein–protein interaction networks were generated using STRING (version 12.0) [48]. Networks were partitioned using STRING *k*-means clustering, using the minimum recommended number of clusters (*k* = 9 for miR-30e-3p and *k* = 11 for miR-34b-5p) allowing interpretable clusters without excessive fragmentation. Functional enrichment analysis was performed using the Ingenuity Knowledge Base (Ingenuity Pathway Analysis; Qiagen, Redwood City, CA, USA), with *p* < 0.05 and an absolute activation *z*-score ≥ 2, where calculated. Data visualisation was performed in R (version 4.3.1) using ggplot2 [49] and VennDiagram [50].

### 2.10. Apoptosis Protein Array Analysis

Apoptosis-related proteins were assessed using the Proteome Profiler Human Apoptosis Array Kit (R&D Systems, ARY009, Bio-Techne Ltd, Abingdon, UK) according to the manufacturer’s instructions with minor modifications. Briefly, lysates containing 200 µg total protein per sample were incubated with the array membranes overnight at 4 °C with gentle rocking to allow binding of apoptosis-related proteins to their respective capture antibodies. After washing, membranes were incubated sequentially with a cocktail of biotinylated detection antibodies and streptavidin–horseradish peroxidase. Chemiluminescent signals were developed using the kit’s detection reagents and imaged on a digital imaging system. Spot intensities were quantified using ImageJ with the Protein Array Analyzer macro version 1.1c (http://image.bio.methods.free.fr/ImageJ/?Protein-Array-Analyzer-for-ImageJ.html (accessed on 1 April 2025)) mean signal intensities of duplicate spots were background-subtracted and normalised to internal positive controls as recommended by the manufacturer. For heatmap visualisation, normalised signal intensities were natural log(ln)-transformed, and the mean transformed value across the five independent placentas was calculated for each protein and treatment condition. Original unprocessed array membrane images are shown in Supplementary Figure S1.

### 2.11. Statistical Analysis

All statistical analyses were performed using GraphPad Prism version 9.3.1. Data were analysed using non-parametric, paired tests unless otherwise stated. For normalised datasets, Wilcoxon signed-rank tests were used to assess differences relative to control values. System A amino acid transport data were analysed by linear regression, with comparisons of slopes and intercepts/elevations between experimental groups. Statistical significance was set at *p* < 0.05. For apoptosis array data, differences between miRNA inhibitor and matched NT control conditions were assessed using multiple paired *t*-tests with correction for false discovery rate.

## 3. Results

### 3.1. Establishment of Ex Vivo and In Vitro Models of Placental Folate Deficiency

**Figure 1:**
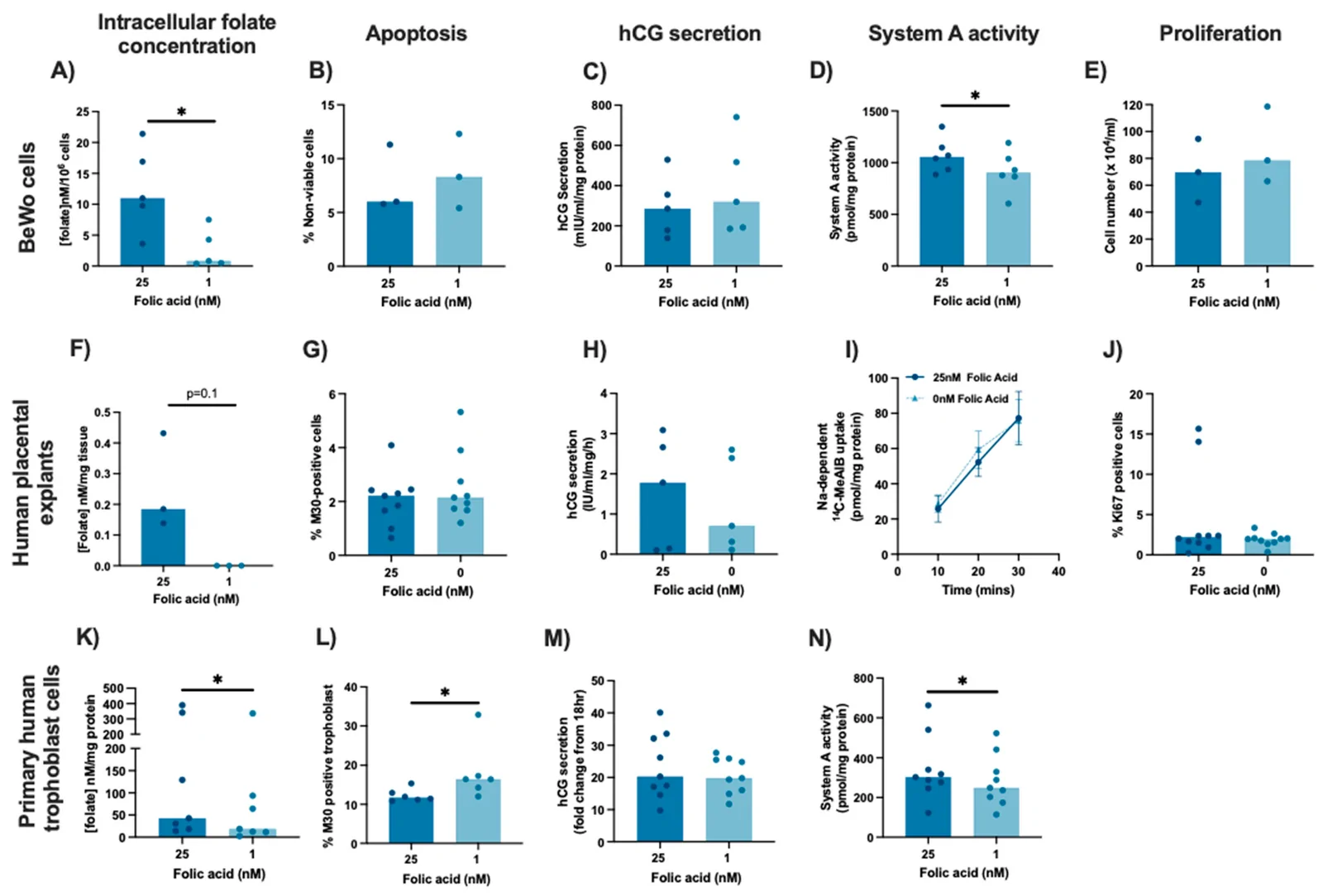
Model-dependent effects of folate deficiency on trophoblast function. The effects of folate-deficient culture conditions were assessed in (**A**–**E**) BeWo cells, (**F**–**J**) placental villous explants and (**K**–**N**) primary human cytotrophoblasts. (**A**–**E**) BeWo cells: (**A**) intracellular folate concentration after 4 days of culture (*n* = 5 passages); (**B**) levels of non-viable cells assessed by incorporation of Trypan blue; (**C**) hCG secretion measured by ELISA; (**D**) system A amino acid transport activity measured as Na^+^-dependent uptake of ^14^C-MeAIB at 30 min (*n* = 6 passages); and (**E**) cell viability assessed by levels of trypan blue exclusion (*n* = 3 passages). (**F**–**J**) Placental villous explants: (**F**) tissue folate concentration (*n* = 3 placentas); (**G**) apoptosis assessed as the percentage of M30-positive cells relative to total nuclei; (**H**) hCG secretion measured by ELISA; (**I**) system A amino acid transport activity measured as Na^+^-dependent ^14^C-MeAIB uptake over time (*n* = 5 placentas); and (**J**) proliferation assessed as the percentage of Ki67-positive cells relative to total nuclei (*n* = 10 placentas). (**K**–**N**) Primary human cytotrophoblasts: (**K**) intracellular folate concentration after 114 h of culture (*n* = 7 placentas); (**L**) apoptosis assessed as % M30-positive cells relative to total nuclei; (**M**) hCG secretion measured by ELISA and expressed as fold change relative to 18 h; and (**N**) system A amino acid transport activity measured as Na^+^-dependent uptake of ^14^C-MeAIB at 30 min (*n* = 9 placentas). Bars represent median values and individual points represent independent passages or placentas, as indicated. Pairwise comparisons between folate conditions were performed using the Wilcoxon matched-pairs signed-rank test. For the villous explant system A time-course (**J**), data are presented as mean ± SEM and analysed by linear regression. * *p* < 0.05.

To examine the direct effects of folate deficiency on placental function, intracellular folate depletion was established in BeWo choriocarcinoma cells, human villous explants, and primary human cytotrophoblasts. In BeWo cells, culture in low-folate medium (1 nM) reduced intracellular folate concentrations by >90% (*p* < 0.05) compared with physiological conditions (25 nM) (Figure 1A), and depletion was sustained during prolonged culture. Despite this, folate deficiency did not affect apoptosis, hCG secretion or cell proliferation, (Figure 1B,C,E). System A amino acid transport was unchanged during short-term culture but was modestly reduced after prolonged exposure (12 days; ~15% decrease, *p* < 0.05; Figure 1D). In villous explants, culture under folate-deficient conditions (0 nM) reduced tissue folate to near the limit of detection, although this did not reach statistical significance (Figure 1F). No differences were observed in apoptosis, hCG secretion, system A activity or proliferation between conditions (Figure 1G–J). In contrast, primary cytotrophoblasts cultured in low-folate medium (1 nM) exhibited a significant reduction in intracellular folate (56% decrease, *p* < 0.05; Figure 1K), accompanied by increased apoptosis (*p* < 0.05; Figure 1L) and reduced system A amino acid transport (~18% decrease, *p* < 0.05; Figure 1N), while hCG secretion was unchanged (Figure 1M). Proliferation was not assessed in primary cytotrophoblast as cells undergo terminal differentiation in culture. Together, these data demonstrate that primary trophoblasts most closely reproduced the functional features of placental folate deficiency observed in vivo and were therefore selected for subsequent mechanistic studies [21].

### 3.2. Identification of Folate-Sensitive microRNAs in Primary Trophoblasts

**Figure 2:**
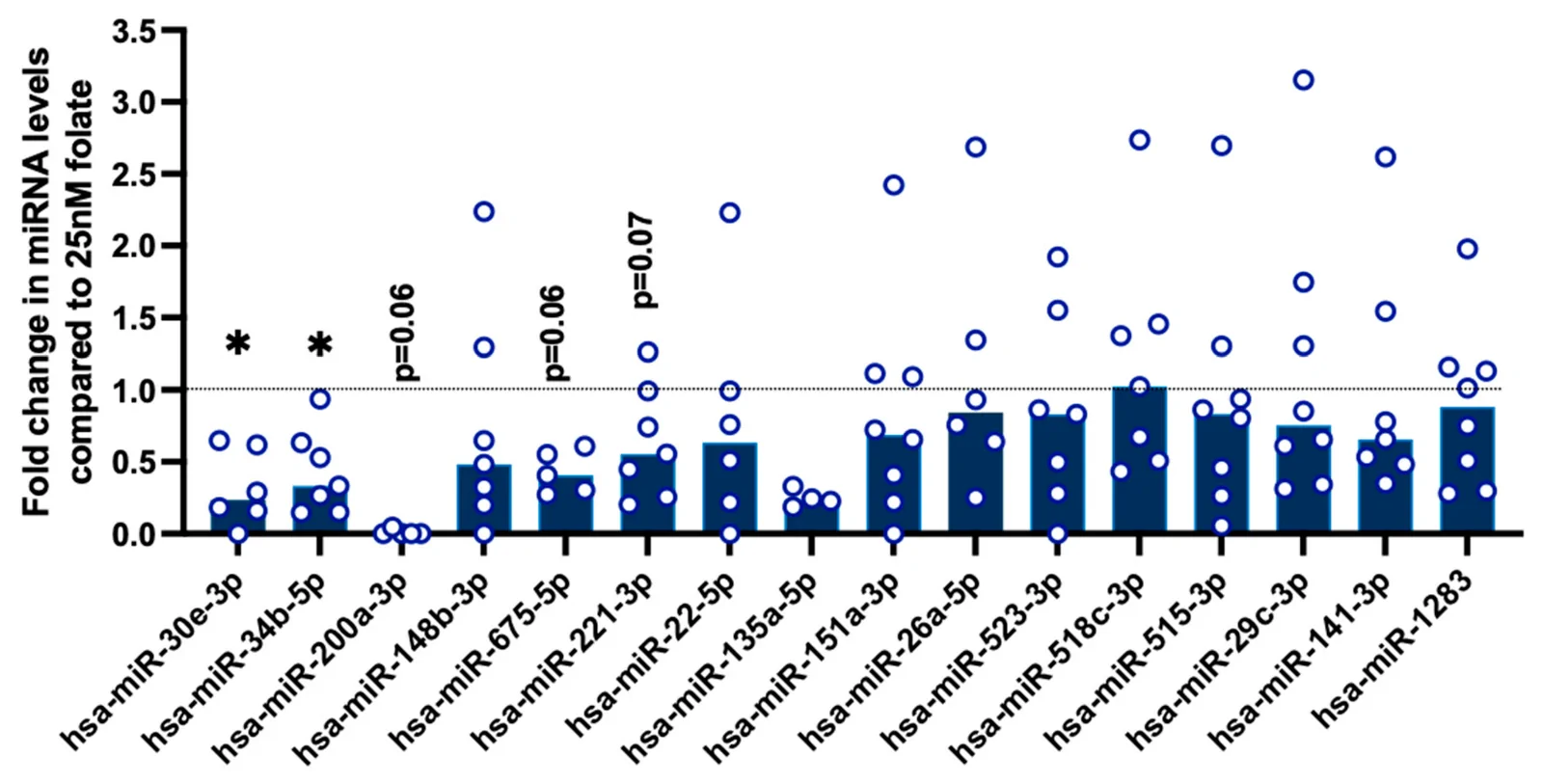
Identification of folate-sensitive miRNAs in primary trophoblasts. Relative expression of miRNAs in trophoblasts cultured under folate deficiency (1 nM) compared to physiological folate (25 nM) were determined by QPCR using specific primer sets. Expression was normalised to U6snRNA and compared to matched controls (25 nM folate). Individual points represent independent placental preparations (*n* = 5–8) and bars represent median values. Statistical significance was determined using the Wilcoxon signed-rank test; * *p* < 0.05 was considered significant.

Sixteen placental microRNAs previously associated with low maternal folate status [21] were assessed in primary trophoblasts cultured under physiological and folate-deficient conditions (Figure 2). Of these, miR-30e-3p (76% reduction, *p* = 0.03) and miR-34b-5p (67% reduction, *p* = 0.016) were significantly reduced under folate-deficient conditions. Whilst there were trends for alteration in three other miRNAs (miR-675-5p, miR-221-3p, and miR-200a-3p; *p* = 0.06–0.07), no other miRNAs showed significant changes. miR-200a-3p and miR-135a-5p were detected at very low abundance in primary cytotrophoblasts, with Ct values frequently >35, so their relative expression should therefore be interpreted cautiously. Overall, these findings indicate that only a subset of folate-associated placental miRNAs showed a robust response to reduced folate availability in primary cytotrophoblasts cultured in vitro.

### 3.3. Folate-Sensitive miRNAs Do Not Regulate Trophoblast Hormone Secretion, System A Activity or Apoptosis

**Figure 3:**
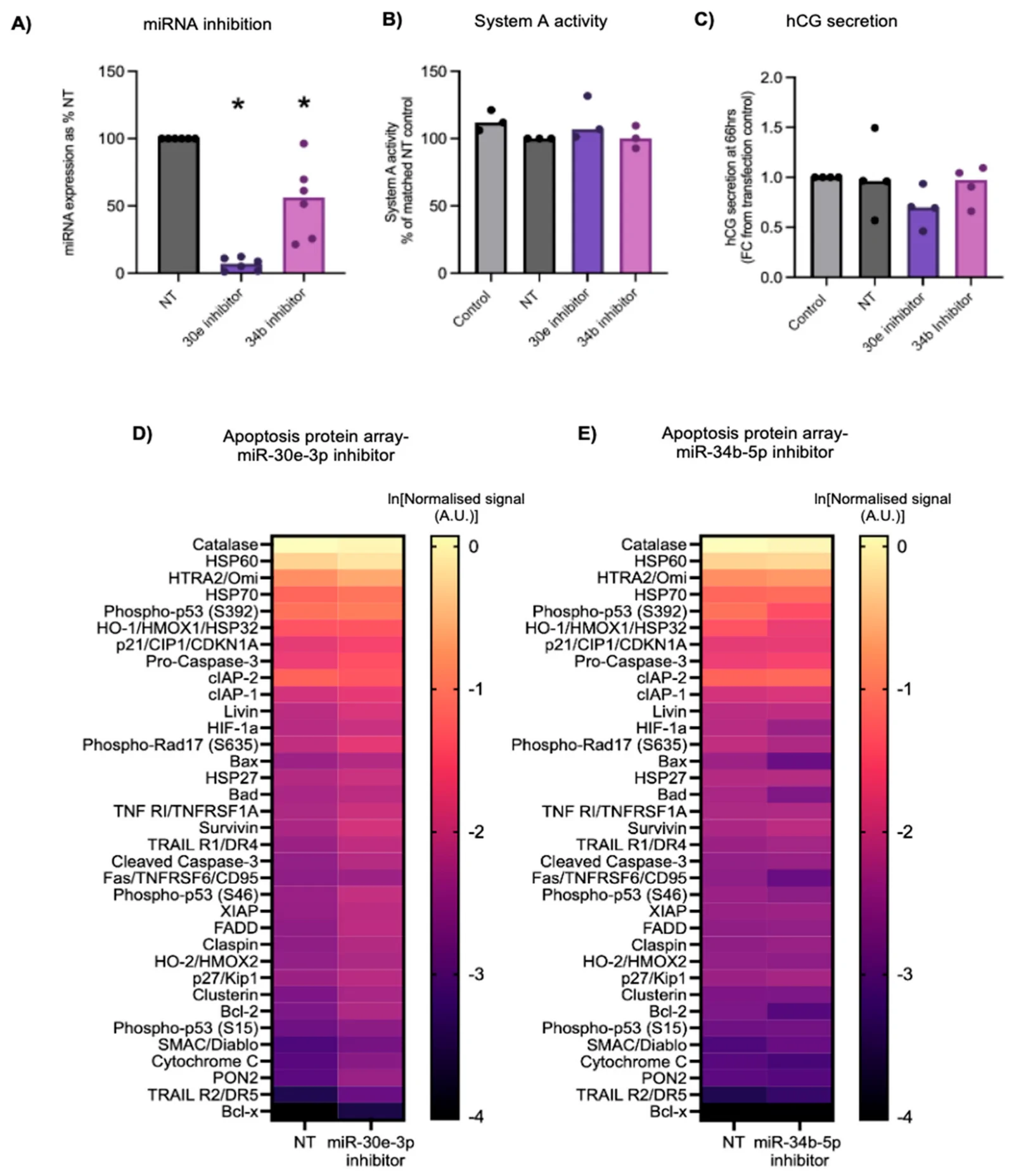
Functional effects of miR-30e-3p and miR-34b-5p inhibition in primary human cytotrophoblasts. (**A**) miR-30e-3p and miR-34b-5p expression 48 h following transfection with specific miRNA or non-targeting (NT) inhibitors (40 nM). (**B**) System A amino acid transport activity (Na^+^-dependent ^14^C-MeAIB uptake) following transfection with reagent alone (control), NT, miR-30e-3p inhibitor (30e) or miR-34b-5p inhibitor (34b), expressed relative to matched NT controls (*n* = 3 placentas). (**C**) β-hCG secretion following transfection, expressed as fold change relative to 18 h (*n* = 4 placentas). (**D**,**E**) Apoptosis-related protein expression following inhibition of (**D**) miR-30e-3p or (**E**) miR-34b-5p compared with NT controls (*n* = 5 placentas). Heatmaps represent mean ln-transformed normalised signal intensities, with colour intensity indicating relative protein abundance. Each data point represent cells isolated from an independent placenta and bars indicate median values in (**A**–**C**). * *p* < 0.05 was considered significant.

To determine whether reductions in miR-30e-3p or miR-34b-5p contribute to functional changes observed under folate-deficient conditions, primary trophoblasts were transfected with specific inhibitors. Inhibition resulted in a reduction in miR-30e-3p (93%) and miR-34b-5p (44%) expression compared with non-targeting controls (*p* < 0.05; Figure 3A). However, inhibition of either miRNA had no effect on system A amino acid transport (Figure 3B) or β-hCG secretion (Figure 3C). Similarly, profiling of apoptosis-related proteins revealed no significant differences following inhibition of either miRNA after correction for multiple testing (Figure 3D,E; Supplementary Figure S2).

### 3.4. Transcriptomic Changes Following miR-30e-3p and miR-34b-5p Inhibition

**Figure 4:**
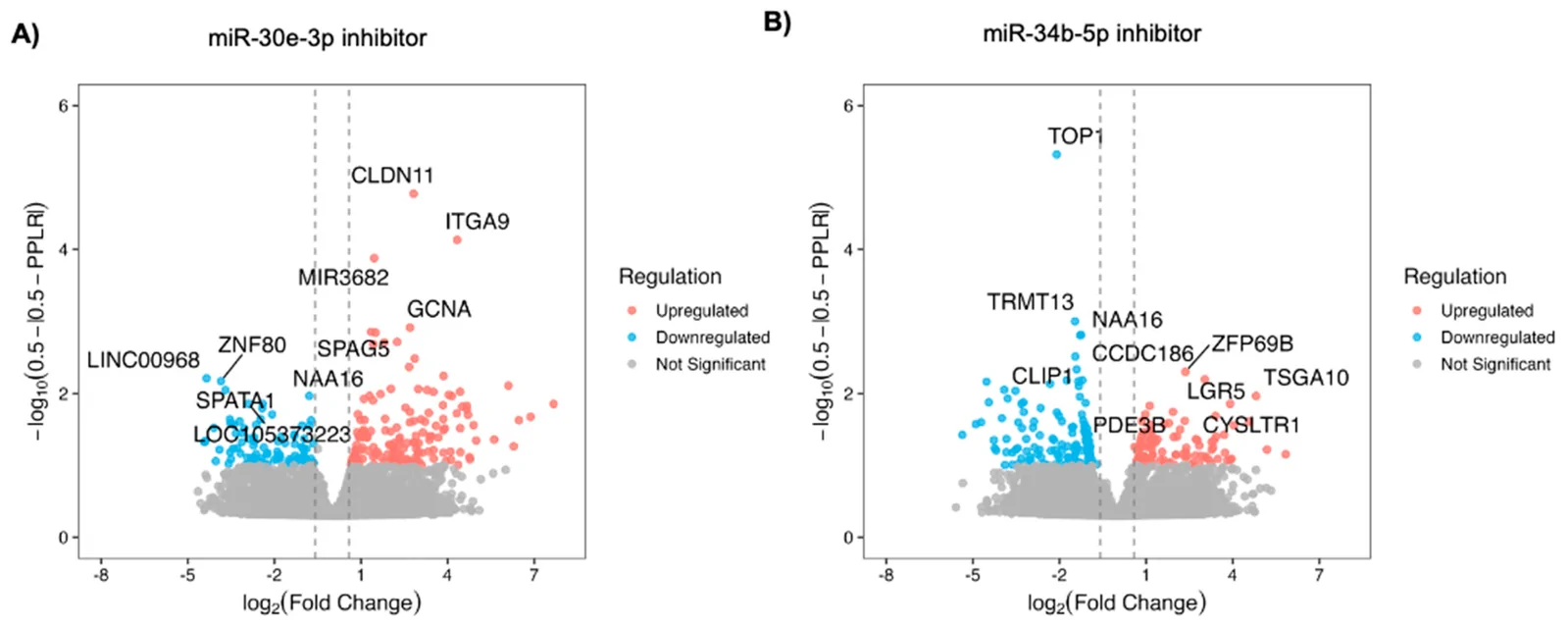
Differential gene expression analysis following miR-30e-3p and miR-34b-5p inhibition in primary trophoblasts. Volcano plots showing differentially expressed genes in primary trophoblast 48 h post-transfection with either (**A**) miR-30e-3p inhibitor or (**B**) 34b-5p inhibitor compared to non-targeting control. Log2 fold change is plotted against the −log10-transformed minimum tail probability derived from the probability of positive log ratio (PPLR), with higher values indicating greater certainty of differential expression. Genes with PPLR > 0.9 were classified as significantly upregulated and those with PPLR < 0.1 as significantly downregulated, and are shown in red and blue, respectively; genes not meeting these thresholds are shown in grey. The five most upregulated and five most downregulated genes are labelled. Dashed vertical lines indicate the fold-change thresholds used to define differential expression.

**Figure 5:**
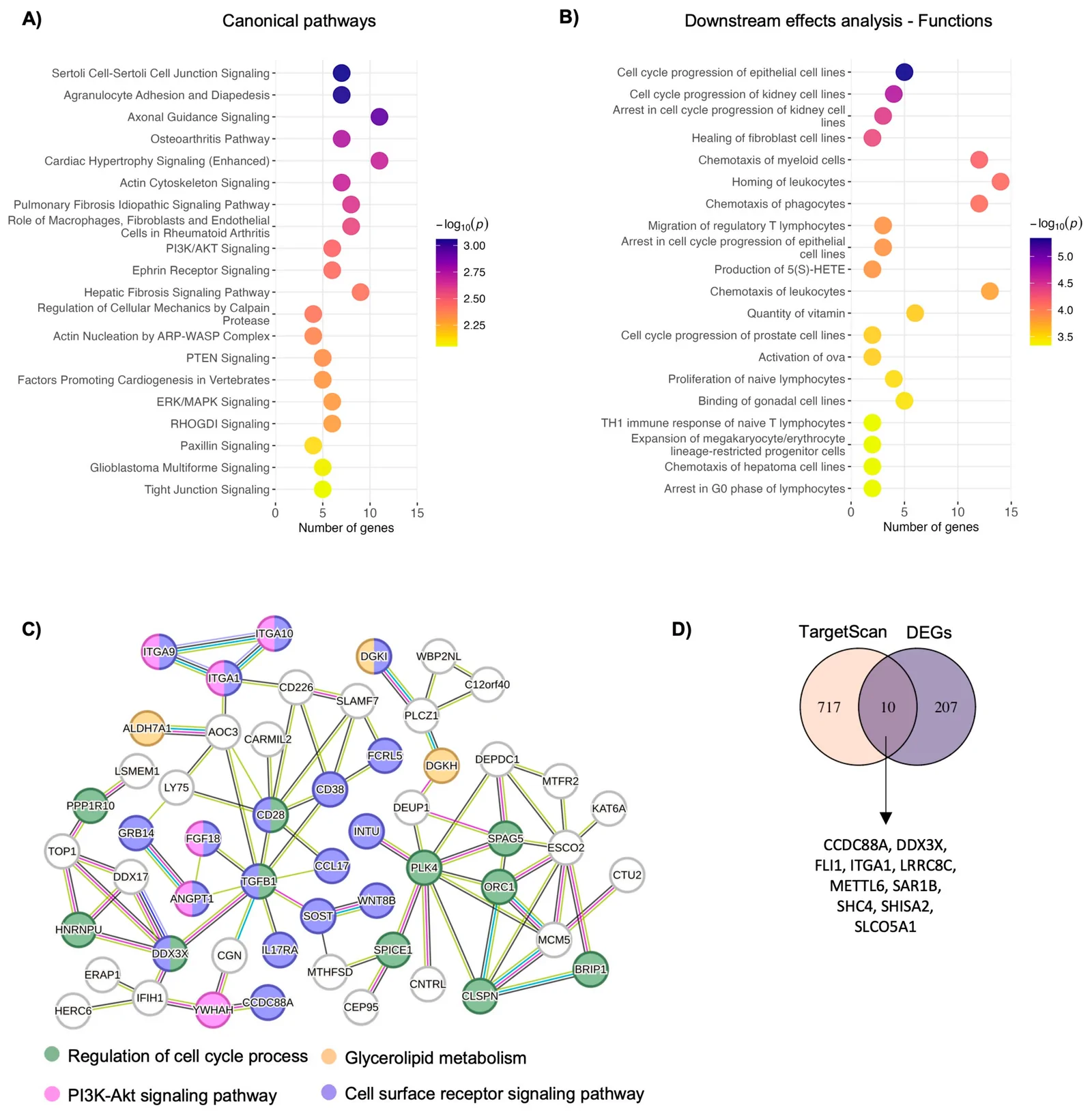
Functional enrichment analyses of genes altered following miR-30e-3p inhibition. Functional predictions were performed using Ingenuity Pathway Analysis of the top 20 (**A**) canonical pathways and (**B**) biological functions sorted by *p*-value. The x-axis represents the number of genes involved in each term (y-axis) and the bubble colour indicates the negative log10 transformation of the *p*-value, with darker colour representing a lower *p*-value. (**C**) Clustering of the network of protein-coding differentially expressed genes using *k*-means in STRING identified 9 clusters with the largest cluster being associated with regulation of cell cycle process (green), glycerolipid metabolism (orange), cell surface receptor signalling pathway (blue), and PI3K-Akt signalling pathway (pink). (**D**) Overlap between TargetScan-predicted miRNA targets and differentially expressed genes. Genes in the intersection are listed. DEGs; differentially expressed genes.

To explore potential regulatory pathways associated with folate-responsive miRNAs, transcriptomic profiling was performed following inhibition of miR-30e-3p or miR-34b-5p. Inhibition of miR-30e-3p resulted in a set of 236 DEGs being identified (Figure 4A; Supplementary Table S2). Canonical pathway analysis identified enrichment of pathways predominantly related to cytoskeletal organisation, cell adhesion and intracellular signalling, including actin cytoskeleton, PI3K/AKT, ERK/MAPK, PTEN, paxillin, ephrin receptor and cell–cell junction signalling (Figure 5A). Biological function analysis additionally identified processes associated with cell cycle regulation, cell migration and immune-cell signalling (Figure 5B). STRING analysis demonstrated extensive protein–protein interactions among the DEGs, with functional annotation identifying networks related to immune processes, cell cycle regulation, PI3K–AKT and WNT signalling (Figure 5C). Integration with TargetScan-predicted miR-30e-3p targets identified 10 DEGs as predicted miR-30e-3p targets (Figure 5D). Upstream regulator analysis additionally identified several predicted regulators of the transcriptional response, including L-homocysteine, which was predicted to be activated following miR-30e-3p inhibition (Supplementary Figure S3A).

**Figure 6:**
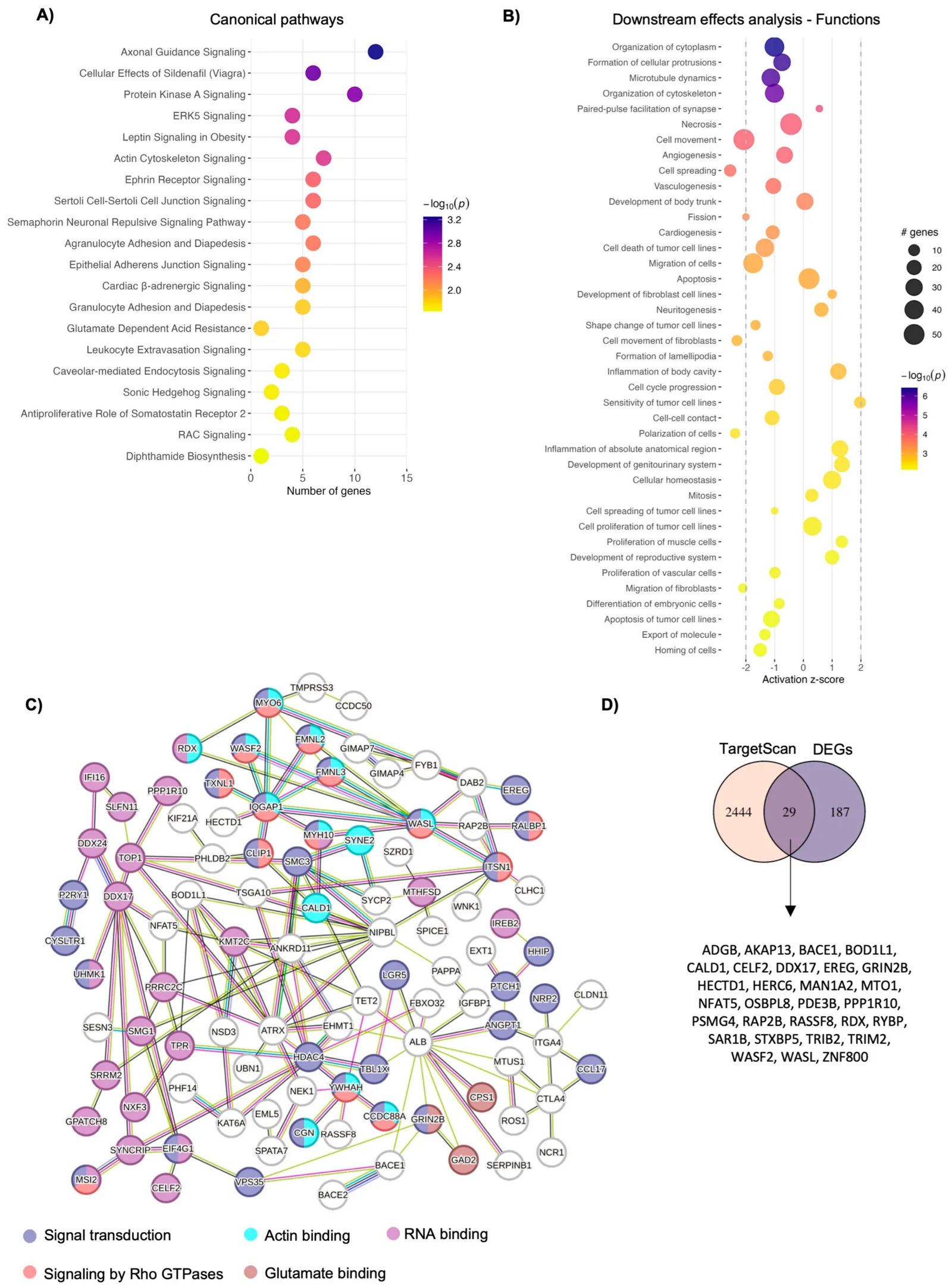
Functional enrichment analyses of genes altered following miR-34b-5p inhibition. Functional predictions were performed using (**A**,**B**) Ingenuity Pathway Analysis of (**A**) the top 20 canonical pathways sorted by *p*-value. The x-axis represents the number of genes involved in each term (y-axis) and the bubble colour indicates the negative log10 transformation of the *p*-value, with darker colour representing a lower *p*-value. (**B**) Biological functions with calculated *z*-scores. The number of genes per term (#genes) are reflected by the size of the bubble. The bubble colour indicates the negative log10 transformation of the *p*-value, with darker colour representing a lower *p*-value. The x-axis indicated the activation where a *z*-score ≥ 2 or ≤−2 is considered significant (inhibited or activated, respectively). Dashed lines represent the *z*-score cutoff. (**C**) Clustering of the network of protein-coding differentially expressed genes using *k*-means in STRING identified 11 clusters with the largest cluster being associated with regulation of signal transduction (dark blue), signalling by Rho GTPases (red), actin binding (pale turquoise), glutamate binding (brown), and RNA binding (purple). (**D**) Overlap between TargetScan-predicted miRNA targets and differentially expressed genes. Genes in the intersection are listed. DEGs; differentially expressed genes.

Inhibition of miR-34b-5p produced a distinct transcriptomic profile (Figure 4B; Supplementary Table S3), comprising 225 differentially expressed genes (DEGs). Canonical pathway analysis identified enrichment of pathways related predominantly to cytoskeletal organisation, cell adhesion and intracellular signalling, including actin cytoskeleton, ephrin receptor and cell–cell junction signalling (Figure 6A). Consistent with this, biological function analysis identified processes associated with cytoskeletal organisation, cell movement, adhesion and cellular organisation (Figure 6B). STRING analysis demonstrated extensive protein–protein interactions among the DEGs, with functional annotation identifying networks related to signal transduction, Rho GTPase signalling and actin binding, alongside RNA- and glutamate-binding functions (Figure 6C). Integration with TargetScan-predicted miR-34b-5p targets identified 29 DEGs as predicted targets of miR-34b-5p (Figure 6D), representing candidate genes through which miR-34b-5p may contribute to the observed transcriptomic changes. Upstream regulator analysis identified a distinct set of predicted regulators following miR-34b-5p inhibition (Supplementary Figure S3B). GSEA of the complete ranked transcriptomic datasets against the MSigDB Hallmark gene-set collection identified no significantly enriched gene sets following inhibition of either miR-30e-3p or miR-34b-5p after correction for multiple testing (FDR-adjusted *p* > 0.05; Supplementary Table S4). Similarly, chi-square analysis showed no significant enrichment of TargetScan predicted miRNA targets within either the miR-30e-3p or miR-34b-5p DEG dataset (miR-30e-3p: χ^2^(1) = 0.85, *p* = 0.357; miR-34b-5p: χ^2^(1) = 0.14, *p* = 0.708).

## 4. Discussion

Low maternal folate status is associated with placental dysfunction and adverse pregnancy outcomes, including impaired fetal growth [21]. In this study we demonstrate that folate deficiency directly impairs trophoblast survival and amino acid transport, independent of maternal or fetal influences. Furthermore, we show that only a subset of placental miRNAs previously associated with low maternal folate status responds directly to folate depletion, and that inhibition of these individual folate-responsive miRNAs is insufficient to reproduce the trophoblast phenotype. Together, these findings suggest that placental adaptation to folate deficiency is mediated through coordinated nutrient-sensitive molecular networks rather than individual miRNAs acting in isolation. These findings are consistent with in vivo and in vitro studies [22,25,27], and provide mechanistic insight into how reduced folate availability contributes to placental dysfunction.

A further key finding was that primary human cytotrophoblasts most closely reproduced the functional features of placentas from folate-deficient pregnancies. Despite effective intracellular folate depletion in all models, only primary trophoblasts exhibited both increased apoptosis and reduced system A activity and were therefore selected for mechanistic studies. This likely reflects differences in metabolic regulation and stress responsiveness between transformed trophoblast cell lines [51,52], multicellular explant tissue and primary cells, highlighting the importance of model selection when studying nutritional regulation of placental function [53]. Nevertheless, BeWo cells and placental explants remain valuable complementary models for investigating other aspects of folate biology and placental physiology that were not addressed in the present study.

Previous human and animal studies have identified associations between maternal folate status and altered placental miRNA expression but did not distinguish whether the observed changes were a direct consequence of reduced folate availability or reflected secondary adaptations within the placenta. Here only miR-30e-3p and miR-34b-5p were significantly reduced under folate-deficient conditions in primary trophoblasts. However, targeted inhibition of either miRNA did not reproduce the effects of maternal folate deficiency on trophoblast apoptosis, amino acid transport or endocrine function. To our knowledge, this is the first study to demonstrate experimentally that only a subset of folate-associated placental miRNAs responds directly to folate depletion and that inhibition of individual folate-responsive miRNAs is insufficient to reproduce the trophoblast phenotype. This distinction between association and functional contribution suggests that coordinated changes across multiple miRNAs and other nutrient-sensitive regulatory networks are likely responsible for trophoblast responses to folate deficiency [54,55,56,57,58].

Although inhibition of either miRNA did not reproduce the functional effects of folate deficiency, transcriptomic analyses identified broader molecular changes that may provide insight into their biological roles. Pathway analyses following miR-30e-3p and miR-34b-5p inhibition predominantly implicated cytoskeletal organisation, cell adhesion and intracellular signalling, including PI3K/AKT, MAPK integrin-associated and Rho GTPase signalling. These pathways regulate trophoblast differentiation, migration, adhesion and syncytialisation and are important for placental development and adaptation across gestation [59,60,61]. Thus, folate-responsive miRNAs may influence broader aspects of trophoblast organisation and signalling that were not captured by the apoptosis, amino acid transport and endocrine endpoints examined here.

Integration of the transcriptomic datasets with predicted miRNA targets identified 10 and 29 DEGs as predicted targets of miR-30e-3p and miR-34b-5p, respectively. Predicted targets were not significantly enriched among the DEGs, indicating that the broader transcriptional response to inhibition of either miRNA was not dominated by predicted direct targets. Consistent with this, GSEA of the complete ranked transcriptomic datasets did not identify significantly enriched Hallmark gene sets following inhibition of either miRNA. Together, these findings suggest that inhibition of individual folate-responsive miRNAs produces relatively modest and dispersed transcriptomic effects rather than coordinated regulation of a dominant gene set or pathway. Nevertheless, the overlapping genes provide candidate miRNA–mRNA relationships for future experimental validation. The relatively limited overlap also suggests that many of the observed transcriptomic changes may represent indirect or downstream consequences of miRNA inhibition. Interestingly, miR-30e-3p has previously been identified as part of a B-vitamin-responsive miRNA–mRNA regulatory network in amniocytes from first-trimester neural tube defect pregnancies [58], providing independent evidence linking nutrient-sensitive miRNA regulation with molecular pathways important for placental and fetal development.

Homocysteine emerged from miR-30e-3p pathway analysis as a predicted upstream regulator, providing a potential link between folate-sensitive miRNA regulation and one-carbon metabolism. Elevated homocysteine is a recognised biochemical marker of impaired one-carbon metabolism [1] and is associated with trophoblast cytotoxicity in vitro and impaired fetal growth, pre-eclampsia, and reduced placental weight in vivo [3,62,63]. However, homocysteine was not directly measured here, and upstream regulator analysis is predictive rather than evidence of a direct mechanistic relationship. These findings therefore raise the possibility that miR-30e-3p participates in a broader regulatory network linking folate availability, one-carbon metabolism and trophoblast function.

Several limitations should be acknowledged. First, experiments were performed using term primary trophoblasts, whereas folate deficiency is likely to exert particularly important effects during early placental development. Second, only two folate-responsive miRNAs were functionally interrogated, and inhibition of individual miRNAs may not recapitulate the coordinated changes in miRNA networks that occur under folate-deficient conditions. Third, isolated trophoblasts do not capture the cellular complexity of the placenta. miR-200a-3p and miR-135a-5p showed very low abundance in primary cytotrophoblasts (Ct > 35), limiting reliable assessment of their response to folate depletion and raising the possibility that folate-associated miRNA responses may differ between placental cell populations. Finally, transcriptomic findings were not independently validated and should be considered hypothesis-generating.

Future studies should investigate coordinated regulation by multiple folate-responsive miRNAs together with upstream epigenetic mechanisms, including DNA methylation and one-carbon metabolism. It will also be important to determine whether high maternal folate exposure influences placental miRNA expression and function, as both folate deficiency and excess may alter placental development through distinct molecular mechanisms. Extending these studies to first-trimester trophoblasts, placental organoids and multicellular placental models will provide greater insight into how folate availability regulates placental development throughout gestation and whether folate-responsive miRNA networks are differentially regulated across distinct placental cell populations. Understanding the molecular mechanisms by which altered maternal folate status influences placental function may help explain why adverse pregnancy outcomes remain associated with inadequate folate status despite widespread folic acid supplementation recommendations and could ultimately inform strategies to optimise placental development and fetal growth.

## 5. Conclusions

This study provides new mechanistic insight into how folate deficiency alters trophoblast biology and demonstrates that placental responses to reduced folate availability are regulated through coordinated nutrient-sensitive molecular networks rather than individual miRNAs. These findings provide a foundation for future studies investigating how altered maternal folate status influences placental development and fetal growth.

## Data Availability

The original microarray data presented in the study are openly available in the NCBI GEO repository at https://www.ncbi.nlm.nih.gov/geo/ under accession no. GSE217721.

## Supplementary Materials

The following supporting information can be downloaded at: https://www.mdpi.com/article/10.3390/nu18172785/s1, Figure S1: Unprocessed blot images from Apoptosis Proteome Profiler array data; Figure S2: Apoptosis Array quantification; Figure S3: Ingenuity Pathway Analysis of predicted master regulators following miRNA inhibition; Table S1: Target sequences of LNA miRNA primer sets utilised for Q-PCR; Table S2: DEGs identified in trophoblast transfected with miR-30e-3p inhibitor; Table S3: DEGs identified in trophoblast transfected with miR-34b-5p inhibitor; Table S4: GSEA of the complete ranked gene-level transcriptomic dataset from trophoblast transfected with miR-30e-3p- or miR-34b-5p inhibitors against the MSigDB Hallmark gene-set collection.

## Abbreviations

The following abbreviations are used in this manuscript:

AKT	AKT serine/threonine kinase 1
BIM	Bcl-2-like protein 11
DEG	differentially expressed genes
ERK	extracellular signal-regulated kinase
FA	folic acid
FBS	fetal bovine serum
FDR	false discovery rate
FFPE	formalin-fixed paraffin embedded
FOXO1	forkhead box O1
GSEA	gene set enrichment analysis
hCG	human chorionic gonadotropin
IBC	individualised birthweight centile
ITGA	integrin alpha subunit
MAPK	mitogen-activated protein kinase
14C-MeAIB	14C-methylaminoisobutyric acid
miRNA	microRNA
NTC	non-targeting control
NTD	neural tube defects
PI3K	phosphatidylinositol 3-kinase
PPLR	probability of positive log ratio
PTEN	phosphatase and tensin homolog
PUMA	p53 upregulated modulator of apoptosis
RAP2B	RAS-related protein 2b
SLC38A1	solute carrier family 38 member 1
STRING	Search Tool for the Retrieval of Interacting Genes/Proteins
WHO	World Health Organisation
WNT	wingless-related integration site
