# Supplementary material for: Folate Deficiency Alters microRNA Expression and Transcriptomic Networks in a Human Trophoblast Model
Source: Nutrients. 2026 Aug 26;18(17):2785. doi: 10.3390/nu18172785 (PMC13567296; doi:10.3390/nu18172785)
Supplement: Supplementary file 1 [file nutrients-18-02785-s001.zip › Supplementary Table S1_miRNA primers.pdf]

Table S1. Target sequences of LNA miRNA primer sets utilised for Q-PCR.

miRNA ID	Primer target sequence (5’-3’)	Accession No.
**hsa-miR-675-5p**	UGGUGCGGAGAGGGCCCACAGUG	MIMAT0004284
**hsa-miR-523-3p**	GAACGCGCUUCCCUAUAGAGGGU	MIMAT0002840
**hsa-miR-518c-3p**	CAAAGCGCUUCUCUUUAGAGUGU	MIMAT0002848
**hsa-miR-515-3p**	GAGUGCCUUCUUUUGGAGCGUU	MIMAT0002827
**hsa-miR-30e-3p**	CUUUCAGUCGGAUGUUUACAGC	MIMAT0000693
**hsa-miR-34b-5p**	UAGGCAGUGUCAUUAGCUGAUUG	MIMAT0000685
**hsa-miR-221-3p**	AGCUACAUUGUCUGCUGGGUUUC	MIMAT0000278
**hsa-miR-29c-3p**	UAGCACCAUUUGAAAUCGGUUA	MIMAT0000681
**hsa-miR-26a-5p**	UUCAAGUAAUCCAGGAUAGGCU	MIMAT0000082
**hsa-miR-22-5p**	AGUUCUUCAGUGGCAAGCUUUA	MIMAT0004495
**hsa-miR-200a-3p**	UAACACUGUCUGGUAACGAUGU	MIMAT0000682
**hsa-miR-151a-3p**	CUAGACUGAAGCUCCUUGAGG	MIMAT0000757
**hsa-miR-148b-3p**	UCAGUGCAUCACAGAACUUUGU	MIMAT0000759
**hsa-miR-141-3p**	UAACACUGUCUGGUAAAGAUGG	MIMAT0000432
**hsa-miR-135a-5p**	UAUGGCUUUUUAUUCCUAUGUGA	MIMAT0000428
**hsa- miR-1283**	UCUACAAAGGAAAGCGCUUUCU	MIMAT0005799
